# Pandemic transit: examining transit use changes and equity implications in Boston, Houston, and Los Angeles

**DOI:** 10.1007/s11116-022-10345-1

**Published:** 2022-10-28

**Authors:** Julene Paul, Brian D. Taylor

**Affiliations:** UCLA Institute of Transportation Studies, 3320 Public Affairs Building, Los Angeles, CA 90095 USA

**Keywords:** Public transit, Post-pandemic travel, Transportation equity, Neighborhood demographics

## Abstract

While the COVID-19 pandemic upended many aspects of life as we knew it, its effects on U.S. public transit were especially dramatic. Many former transit commuters began to work from home or switched to traveling via private vehicles. But for those who continued to work outside the home and could not drive—who were more likely low-income and Black or Hispanic—transit remained an important means of mobility. However, most transit agencies reduced service during the first year of the pandemic, reflecting reduced ridership demand, increasing costs, and uncertain budgets. To analyze the effects of the pandemic on transit systems and their users, we examine bus ridership changes by neighborhood in Boston, Houston, and Los Angeles from 2019 to 2020. Combining aggregated stop-level boarding data, passenger surveys, and census data, we identify associations between shifting travel patterns and neighborhoods. We find that early in the pandemic, neighborhoods with more poor and non-white households lost proportionally fewer riders; however, this gap between high- and low-ridership-loss neighborhoods shrank as the pandemic wore on. We also model ridership change controlling for multiple factors. Ridership in Houston and LA generally outperformed Boston, with built environment and demographic factors accounting for some of the observed differences. Neighborhoods with high shares of Hispanic and African American residents retained more riders in the pandemic, while those with higher levels of auto access and with more workers able to work from home lost more riders, all else equal. We conclude that transit’s social service role elevated during the pandemic, and that serving travelers in disadvantaged neighborhoods will likely remain paramount emerging from it.

**Supplementary Information:**

The online version contains supplementary material available at 10.1007/s11116-022-10345-1.

## Introduction

The COVID-19 pandemic upended life as we knew it, particularly in cities. As dynamic economic and cultural agglomerations, urban areas facilitate the interactions of people and firms. But during a global public health crisis, the interpersonal proximity enabled by urban living proved not an asset, but a liability. To protect the health of residents and slow the spread of illness before vaccines became widely available, public officials and agencies recommended that people drastically curtail their interactions with others. Public health orders encouraged people to work, study, shop, and socialize from home when possible and to minimize travel. The effects of these orders were especially dramatic early on: from February to April 2020, passenger vehicle travel fell by almost 60 percent in the U.S. (Schuman 2020). And mounting evidence suggests that expanded work-from-home policies have proven popular with both employers and employees and will likely persist at least part-time for many workers post-pandemic (Barrero et al. 2021). These changes could fundamentally change the nature of travel in the U.S. and around the globe.

But while all travel in U.S. cities declined in spring 2020, public transit use was hit especially hard and has proven the slowest to recover. Public transit buses, trains, and vans, which excel at moving large numbers of people in the same direction at the same time, faced unique challenges. Transit requires riders and operators to share space in a manner quite different from private automobile travel. Further, transit disproportionately serves journeys to and from work (Federal Highway Administration 2018), largely in the peak hours, and often into and out of densely populated downtowns. Should downtown office work shift to home, at least part time, over the longer run the effects may prove especially problematic for public transit (Brough et al. 2021).

As travel gradually resumed in the late spring and early summer of 2020, travel modes that enable social distancing—like private cars and trucks, bicycling, and walking—recovered relatively quickly, and by mid-2021 were near or above pre-pandemic levels. Public transit, by contrast, recovered much more slowly (Bureau of Transportation Statistics 2021). After the initial crash in patronage in March and April 2020, transit ridership partially recovered to about one-quarter to three-quarters of pre-pandemic ridership levels, depending on the system and mode. Ridership dipped again amidst the spike in infections during the late fall of 2020 and winter of 2021, before resuming the slow pace of patronage recovery. U.S. transit ridership dipped yet again during the rapid spread of the Omicron variant in the late fall of 2021 and winter of 2022. While ridership again slowly recovered afterward, by April 2022, national transit ridership remained at only 62 percent of pre-pandemic levels (American Public Transportation Association 2022).

Preliminary evidence suggests that riders who remained on public transit throughout the pandemic (or who returned as it wore on) were disproportionately those who could not work from home, did not own a car, and did not lose their jobs (He et al. 2022; Parker et al. 2021). Providing mobility for these more economically precarious travelers is a major role for public transit systems (Garrett and Taylor 1999), and this role was elevated in the pandemic. While transit agencies often aspire to draw auto users out of cars, almost half of transit users lack regular access to a private vehicle (American Public Transportation Association 2017). Serving and attracting such riders, who have fewer transportation and workplace options, will likely remain an important part of transit’s recovery. Further, as federal pandemic relief funds are spent down, the long-term financial stability of U.S. transit will depend on when, where, and to what extent various riders return. This makes urgent an understanding of the contours and predictors of the evolving demand for public transit.

Ridership did not just fall during the pandemic, it did so unevenly across neighborhoods and users. Accordingly, we examine how transit use shifted during the first year of the COVID-19 pandemic, with a focus on built environment and neighborhood socioeconomic characteristics across three very different U.S. metropolitan areas. To do this, we analyze aggregated stop-level weekday boardings on Massachusetts Bay Transportation Authority (MBTA), Houston Metro, and Los Angeles Metro bus services; these are, by far, the largest transit operators in their respective regions. We aggregate these data to census tracts for four periods prior to, at the beginning, and well into the pandemic. To examine transit demand, we identify the neighborhoods that lost more or fewer pre-pandemic riders. We combine these data with built environment and neighborhood socioeconomic data to estimate ordinary least squares (OLS) regression models predicting changes in ridership. This analysis is unique in its spatial granularity across three large metropolitan areas. Our findings indicate that some of the regional differences in ridership change indeed reflect the differing regional built form and neighborhood demographics served by the three agencies, although substantial independent effects across the three regions remain. We also show that bus ridership demand shifted dramatically away from downtowns and other densely populated centers as well as places with higher levels of automobile access and workers who are more likely able to work from home, and toward lower-income and non-white neighborhoods—which collectively elevate public transit’s social service role.

## Previous research

While the COVID-19 pandemic caused all types of travel to initially fall across many different travel modes, due to stay-at-home orders and other public health restrictions, the declines in travel were greater among higher-income people (Brough et al. 2021) who were more likely than lower wage workers to work from home (Jiao and Azimian 2021). These steep drops in ridership were on top of more gradual patronage declines in the years leading up to the pandemic (Berrebi and Watkins 2020; Manville et al. 2022). In addition, because those with access to private vehicles were able to travel about without coming in close contact with strangers, and because vehicle access is positively related to income, those who rode transit early in the pandemic tended to be lower income (and more likely Black or Hispanic, and immigrants) than pre-pandemic transit riders. Detailed data on the behavior of transit users during the pandemic are in short supply, so researchers have tended to either rely on rider surveys that typically do not collect detailed trip data, or on detailed trip data that lack information about travelers. (We rely here on the latter combined with census data, as we explain below).

### Surveys of changes in transit riders

Some recent research has surveyed riders to assess the effect COVID-19 on travel behavior. This approach allows researchers to question respondents on the specific reasons behind any travel behavior changes (or lack thereof). However, such pandemic surveys may suffer from selection bias and thus not accurately represent transit riders at large. Sampling riders has been a particular challenge during the pandemic, which significantly curtailed strategies for recruiting respondents. Many social scientists relied exclusively on web-based tools to survey travelers (Hlatshwako et al. 2021), which can be problematic as lower-income, immigrant, and older travelers tend to have less Internet access than others.

For example, in an early pandemic travel behavior survey conducted by Conway et al. (2020) in the spring 2020, 59 percent of respondents reported having graduate degrees, a figure more than four times higher than the national average (13%). To assess the potential magnitude of this sampling bias, Zhang et al. (2020) examined data on transit users recruited on social media sites. They found that recruiting respondents via Facebook tended to overrepresent certain types of transit riders, including women and people with higher incomes (Zhang et al. 2020). With these representativeness limitations in mind, we review the results of some of these surveys below.

Some researchers have identified changes in patterns of the socioeconomic (SES) characteristics of transit riders prior to versus during the pandemic. Research on pre-pandemic transit rider demographics consistently found that the average transit user in the U.S. was poorer and more likely to be a person of color than all travelers (Federal Highway Administration, 2018). To assess changes in the characteristics of regular transit riders, Parker et al. (2021) conducted a national survey of pre-pandemic transit riders and non-riders in August 2020. Within the category of pre-pandemic transit riders, they also distinguished between low-income (with household incomes under $25,000) and non-low-income ones. They found that the former reduced their transit trips much less than the latter (Parker et al. 2021). He et al. (2022) also surveyed transit users at the national level. They found that U.S. travelers with automobile access were far more likely to reduce their use of public transit than travelers lacking access to autos (He et al. 2022).

Using data from another national survey, Jiao and Azimian (2021) analyzed responses to the U.S. Census Household Pulse Survey (HPS) to compare travel behaviors in September and October of 2020 with the pre-pandemic era. Their results differed slightly from the findings from the two previously mentioned surveys. Like the two other studies, certain factors associated with low SES, including lower incomes and education levels, decreased the likelihood that a traveler reduced their public transit trips, all else equal. However, other factors associated with low SES, including being non-white and reporting difficulty with expenses during the pandemic, increased the likelihood that a traveler reduced their public transit trips (Jiao and Azimian 2021).

Researchers have also identified associations among pandemic transit use, trip purpose, and employment sector. For example, because some jobs can more easily be done remotely than others, research has shown rates of working-from-home early in the pandemic varied substantially from industry to industry (Dingel and Neiman 2020). These relationships among employment sector, working-from-home, and commuting have implications for public transit. Palm et al. (2021) conducted a survey of frequent pre-pandemic transit users in Toronto and asked about their use of transit after the onset of the pandemic in March 2020. So-called essential workers—such as grocery clerks and nurses who have been needed at worksites throughout the pandemic—comprised a significant portion of their sample: 72 percent of retail workers and 55 percent of healthcare and social assistance workers reported continuing to take transit. Further, the decline in transit use varied significantly by trip purpose in their sample: recreation and exercise trips fell 89 percent, work trips by 79 percent, and grocery trips by 60 percent. The authors also concluded that traveler financial constraints played a major role in explaining pandemic transit use (Palm et al. 2021).

### Studies of geographic patterns of transit use change

Other researchers have used ridership data from transit agencies and/or mobile device data to identify relationships between transit ridership and neighborhood characteristics. These studies tend to focus on single agencies or regions. Such studies have identified aggregate ridership patterns and trends in transit by lines, networks, and geographies; they tend to lack information about individual travelers or trips. We discuss the implications of these limitations for our analysis in ″Data and methodological limitations″ Section below.

Using mobile device data, Brough et al. (2021) studied patterns of ridership change in the Seattle metropolitan area through 2020. They found that residents of higher income and education areas tended to switch from public transit to driving far more often than did residents of lower income and education areas. They also identified a negative relationship between neighborhood education/income and transit ridership change (and with travel overall as well). Further, they found that the ability to work from home was a key factor in pandemic transit use, and that observed geographic disparities in transit use persisted as the pandemic wore on (Brough et al. 2021).

Several studies of Chicago have examined pandemic-era ridership changes on the “L” rail transit system in Chicago. For example, Hu and Chen (2021) found that the median income, rates of education, and proportion of non-Hispanic white residents in a neighborhood were all negatively associated with ridership change from 2019 to 2020. They also identified a negative association between commercial land uses, knowledge sector employment, and ridership change (Hu and Chen 2021). Freemark (2021) similarly found that Chicago neighborhoods with a large proportion of non-Hispanic residents lost fewer riders from 2019 to 2020. He also identified a negative association between the number of jobs in a neighborhood and change in “L” riders, possibly confirming the ability-to-work-from-home hypothesis; neither of Freemark’s findings, however, controlled for other neighborhood characteristics. Finally, Osorio et al. (2022) examined both rail and bus ridership in Chicago during the pandemic and found that the ability to work from home likely accounted for most of the ridership loss. Thus, due to greater ability to work from home, neighborhoods with higher average rates of SES among residents—such as rates of education and income—lost more transit riders (Osorio et al. 2022).

### Regional differences

In addition to studies of traveler characteristics and transit use, and of geographies and transit use within regions, a few studies have examined pandemic transit use across regions in the U.S. Across all modes, transit ridership in the U.S. fell 81 percent between April 2019 and April 2020. However, Liu et al. (2020) found that U.S. transit systems in the Northeast, large coastal cities, and college towns experienced steeper drops in ridership from February to May 2020 than agencies in the Midwest and South. Further, regions with smaller shares of transit use before the pandemic lost fewer riders—both proportionally and in total—than regions with the highest levels of use and the largest systems (American Public Transportation Association 2021).

Scholars have reached limited consensus on the reasons behind these regional differences. One reason could be the highly variable rates of COVID-19 infections and associated public health restrictions across cities and regions, particularly early in the pandemic. For example, New York City Transit—by far the largest U.S. transit operator—serves a region that had an early, intense concentration of COVID-19 cases. Its subway ridership fell over 90 percent during the peak of New York region cases in April 2020 (Frost 2020). Other densely populated cities like Washington D.C. and Boston, which each have downtowns well served by robust public transit systems, also saw early infection waves and associated patronage declines of almost 90 percent (American Public Transportation Association 2021). In contrast to these regions (as well as those on the West Coast), cities in the Midwest and South fell under stay-at-home orders later (Liu et al. 2020), and tended to lift them sooner.

**Fig. 1:**
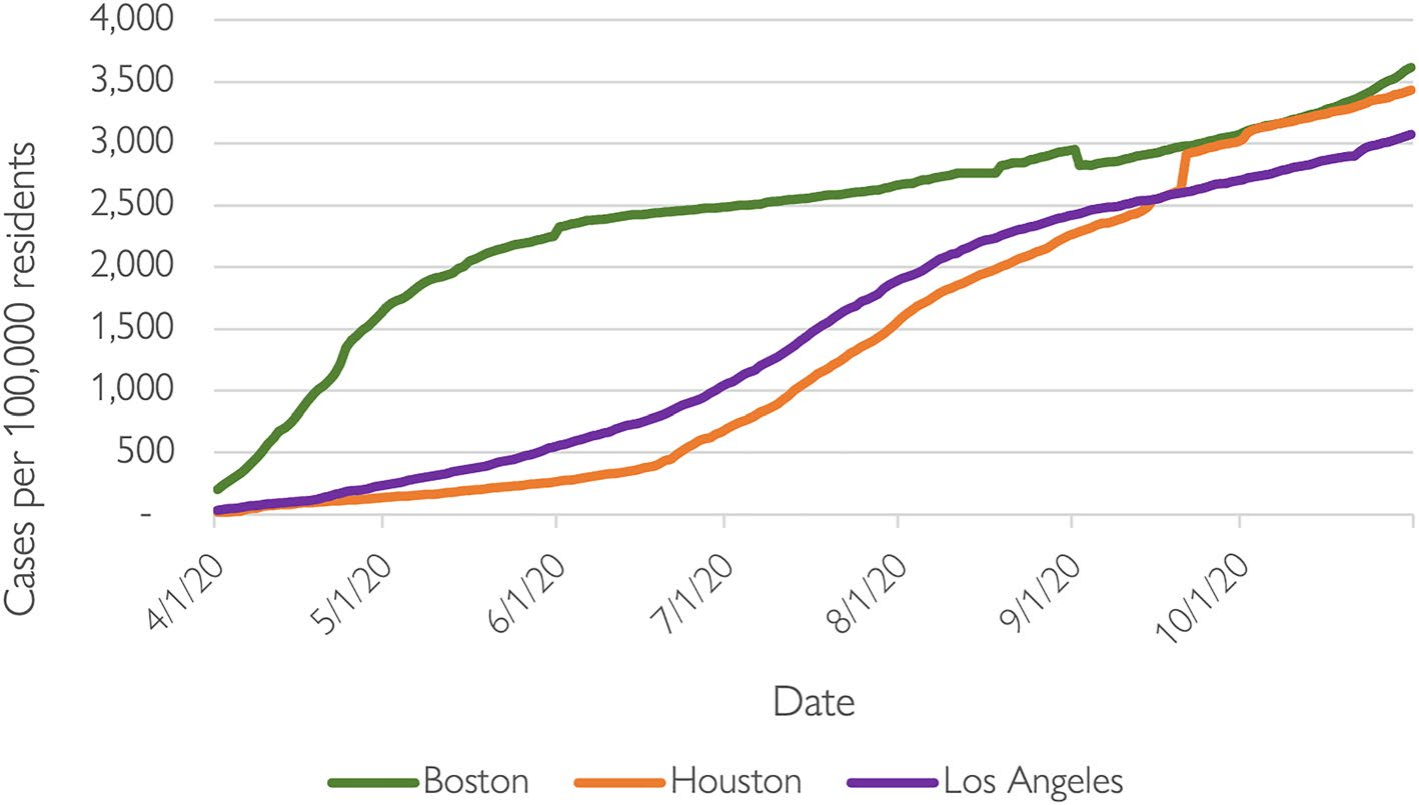
Trends in COVID-19 cases, April 2020 to October 2020. *Data source* The New York Times 2020

Indeed, the three transit agencies we examine here serve regions that had very different patterns of COVID-19 case rates. Figure 1 presents data on the incidence of daily reported COVID-19 cases per 100,000 residents by region. It shows that Boston saw much higher caseloads early in the pandemic. In April 2020, Boston’s average daily rate per 100,000 residents (859) dwarfed that of Los Angeles (121) and Houston (82). By October 2020, however, the gap had closed, with Boston’s rate per 100,000 residents (3,304) similar to Houston’s (3,249), and only slightly higher than in Los Angeles (2,876).

However, beyond the timing of the spread of infection, another determinant of differences in regional transit ridership could reflect the type of service offered in different regions. In general, downtown commuters on rail transit tend to be more educated and well-paid than those traveling by bus to the grocery store (Taylor and Morris 2015). Indeed, transit modes that carried higher shares of more advantaged travelers with access to cars before the pandemic lost a larger share of their riders in the pandemic than modes where more riders had lower incomes and less private vehicle access (Puentes 2020). For example, commuter railroad (−90%) and heavy rail/metro (−87%) ridership collapsed early in the pandemic, while light rail/tram (−74%), bus (−65%), and demand response (−65%) ridership fell less (though still substantially) (American Public Transportation Association 2020). While we primarily examine bus ridership in this study, the role of other modes—particularly the fact that a comparatively large proportion of Boston’s transit riders use the rail system—is an issue we also explore in Sect. 4.2.1 below.

### Gaps in literature

While researchers have gathered information about the drivers of pandemic transit ridership changes, large gaps remain. First, many studies of mobility during COVID-19 have used surveys to explore travel behavior changes. Yet as noted earlier, such studies may suffer from selection bias and not accurately capture the experiences of many transit users as a result. This is particularly true of travelers with less access to the Internet, who are more likely to face economic and transportation challenges. In this analysis we draw on complete boarding data before and during the pandemic, which do not suffer from such sampling bias issues.

Second, most studies of geographically disaggregated transit data examine single regions. In this study we analyze geographically disaggregated boarding data for three very different metropolitan regions. We can thus identify factors associated with ridership changes across all three regions, as well as factors specific to individual regions.

Third, our analysis addresses differences in neighborhood socioeconomic factors within and across regions. Particularly in the multivariate analysis, we test the relative strengths of possible policy and regional effects as well as demographic and built environment effects on ridership at the neighborhood level. Further, by complementing our analysis with rider survey data collected by each agency prior to the pandemic, we can compare how the characteristics of different regional markets might influence ridership response.

Finally, we present both descriptive and multivariate analyses. By simultaneously controlling for many factors, social scientists sometimes fail to represent the experiences of certain groups, especially vulnerable ones who are difficult to reach. While methodologically more sophisticated, multivariate analyses can explain away differences in ways that are not helpful for public policy or planning. Planners may seek to address the transportation challenges that, for example, people living in poverty face, but researchers may find that population density, while correlated with poverty, better predicts behaviors. This can peripheralize the experiences and needs of vulnerable groups. So, in addition to our multivariate analyses, we also describe transit ridership patterns across neighborhoods in our three metropolitan areas to highlight the equity implications for transit managers, urban planners, and public policy makers.

## Data and methods

We describe below the three types of data used, statistical methods employed, as well as the limitations of our approaches.

### Data

We compiled data from multiple sources for three different places to examine rider characteristics and the relationships between bus ridership change and neighborhood characteristics. Our approach has one especially novel aspect: we use stop-level boarding data aggregated to census tracts for three major U.S. transit systems operating in three very different metropolitan environments. Instead of relying on highly aggregated service and ridership data from traditional sources or inferring boardings from scrapes of mobile device data to estimate boardings, we can assess *actual* boarding counts and bus service data at fine levels of spatial aggregation.

#### Ridership data

We analyze bus patronage data from three agencies: the Massachusetts Bay Transportation Authority (MBTA) in Boston, the Metropolitan Transit Authority of Harris County (Houston Metro) in Houston, and the Los Angeles County Metropolitan Transportation Authority (LA Metro) in Los Angeles. These agencies serve three of the largest Combined Statistical Areas (CSAs) in the United States: Los Angeles (#2), Boston (#6), and Houston (#8) (U.S. Census Bureau, 2019a, b). Our study includes data from the largest public transit operator in each of these large regions, which are among the largest public transit agencies in the country as measured by pre-pandemic unlinked passenger trips. In 2019, LA Metro ranked 3rd nationally, MBTA 4th, and Houston Metro 19th (Federal Transit Administration 2020).

The MBTA is the primary transit operator in the greater Boston metropolitan area, serving parts of Essex, Middlesex, Norfolk, Plymouth, and Suffolk counties. We use MBTA stop-level bus service boarding data aggregated to the census tract in which the stop was located, which were compiled and published by the MBTA. With respect to time of day and day of week, we aggregated daily weekday trip counts in a month to arrive at average weekday boardings for the month. We analyze these average weekday boardings for four seasonal time periods: (1) roughly April 2019 (measured from March 25 to April 19, 2019), (2) October 2019, (3) roughly April 2020 (measured from March 23 to April 17, 2020), and (4) October 2020.

Houston Metro is the primary transit operator for Harris County, which contains most of the Houston metropolitan area. We aggregated stop-level bus boarding data provided to us by Houston Metro to census tracts to arrive at average weekday boardings for each tract for four seasonal time periods: (1) April 2019, (2) October 2019, (3) April 2020, and (4) October 2020.

LA Metro is by far the largest transit operator in metropolitan Los Angeles, serving much of Los Angeles County and parts of surrounding counties, although we restricted our analysis to Los Angeles County. Again, LA Metro provided us with stop-level bus boarding data for all their local and rapid bus services that we aggregated to the tract level for the same four months: (1) April 2019, (2) October 2019, (3) April 2020, and (4) October 2020.

In addition to these operator-specific boarding data, we also use National Transit Database (NTD) data to analyze modal- and system-level changes in service and ridership in 2019 to 2020. The NTD differs slightly from our stop-level boarding data in that it reports total ridership by month, as opposed to average weekday boardings. Because agencies report data to the NTD monthly, we can track month-to-month ridership by mode for all months in our study period, and not just for the four months for which we gathered stop-level boardings.

#### Demographic and built environment data

To capture socio-economic and built environment information for the neighborhoods (defined by census tract boundaries) in our sample, we use data from three principal sources.

First, we used the American Community Survey (ACS) 5-year estimates (2014–2018). For each census tract, we assessed the percent of residents who are unemployed and have household incomes below the poverty line. In terms of transportation resources and utilization, we calculated the percentage of workers who commute via transit and the percentage of zero-vehicle households. To account for race/ethnicity, we include data for the percentage of neighborhood (census tract) residents who were non-Hispanic Black/African American, non-Hispanic white, Hispanic, and all others. To control for built environment effects on transit use before and during the pandemic, we also use ACS data on the residential population density of each tract. While the ACS data are samples and not complete counts of the population (compared with the full decennial Census data), our use of the 5-year estimates reduces the sample variance considerably compared to the 1-year estimates.

Second, we used data from the 2019 Longitudinal Employer-Household Dynamics Origin–Destination Employment Statistics (LODES), provided by the Center for Economic Studies at the U.S. Census Bureau. Using these data, we applied a method developed by Dingel and Neiman (2020) to estimate the proportion of employees who can work-from-home, based on *workplace location* in that census tract.

Third, to account for location and centrality of each neighborhood, we use coordinates provided by Manduca (2020) to designate the center of each MSA. We then generated values for each census tract based on their straight-line distance from the central point in each region.

Finally, we also collected monthly COVID-19 caseloads data for the central counties in each of the three regions published by the *New York Times*, which are presented and described above. However, for reasons we explain below, we ultimately excluded these data from the final models presented.

#### Rider surveys

We draw on surveys conducted by two of the transit agencies (MBTA and LA Metro) and Houston's Metropolitan Planning Organization (MPO) to identify systematic differences among bus riders across the different regions. These include: (1) a survey of riders conducted by the MBTA in years 2015–2017; (2) a mid-2017 survey of travelers conducted by the Houston–Galveston Area Council (H-GAC), the MPO serving the greater Houston area; and (3) a survey of riders conducted by LA Metro in fall 2019. These three surveys were conducted to comply with Title VI of the federal Civil Rights Act of 1964 and weighted to reflect the demographics of the full system ridership. They have large sample sizes (about 35,000 for Boston, 20,593 for Houston, and 14,624 for Los Angeles) and low estimated error rates (generally less than 2%).

We use data from these surveys to analyze systematic differences in bus riders across the three regions. While these three surveys utilized different sampling methodologies and occurred in different years, they help contextualize regional differences among bus riders prior to the pandemic.

### Methods

In assessing the relationship between ridership and neighborhood characteristics, we present both descriptive statistics and ordinary least squares (OLS) regression model results. Notably, we test the rates of ridership change, which we define as the change in ridership during the pandemic compared to the most recent, similar pre-pandemic period (e.g., October 2020 boardings were 44% of October 2019 boardings, and thus declined by 56%). We focus on relative rather than absolute changes in boardings because our preliminary analysis indicated that a large share of boardings took place in a relatively small share of tracts. As bus routes typically traverse a large number of tracts, including tracts that host few boardings, we prioritize evaluating how relative shares of ridership and service changed across entire service areas. Other studies of ridership during the pandemic have used this approach, including Liu et al. (2020) and DeWeese et al. (2020).

In the first part of our analysis, we identify census tracts that had the largest and smallest ridership changes at different stages of the pandemic, and then analyze these tracts in light of their neighborhood characteristics. In the second part of our analysis, we model relative changes in census tract level ridership at various stages of the pandemic using OLS regression analysis. We incorporate dummy variables for each region and the socio-economic and built environment variables noted above. We then interact these with the regional dummy variables to identify any unique regional effects beyond those identified from the analysis of the pooled data.

### Data and methodological limitations

Our analysis has limitations. One relates to the ridership data. While the transit agencies whose ridership we analyze are by far the largest in their respective regions, they are not the only transit providers in these areas. For example, Los Angeles County’s other transit operators include Foothill Transit, Long Beach Transit, the Santa Monica Big Blue Bus, and Los Angeles Department of Transportation Commuter Express and Dash bus services, among others. In a neighborhood whose transit users frequently ride the Big Blue Bus, our analysis of LA Metro boardings presents an incomplete picture of transit use changes in that tract. This is a bigger issue in Los Angeles, a region both larger and with more overlapping transit service providers than either Boston or Houston.

MBTA, Houston Metro, and LA Metro also operate rail transit services in conjunction with their bus services, but we only analyze bus—and not all transit—ridership. As we detail below, our focus on relative changes in bus boardings has different implications across the three agencies. The MBTA, in particular, carried a much larger portion—indeed a majority—of pre-pandemic transit trips on rail, as compared with Los Angeles or Houston. To address the partial nature of our data, we focus on percent changes in agency bus boardings in each census tract, rather than absolute changes.

Another issue arises in our associating ridership data with socio-economic data by census tract. As discussed above, we assign the boardings at each bus stop located in each census tract to that tract. But not all bus riders live, work, or complete some activity in the tract where the boarding occurs. Indeed, a non-trivial number of riders may cross tract lines to reach their bus stops. And because census tracts vary in size to reflect differences in population density, travelers may be more likely to cross tract boundaries in central urban areas than in the suburbs. However, given our large sample size of boardings, stops, and tracts, and the clustering of neighborhood characteristics across space, a person who crosses a neighborhood boundary to take a bus likely resides, works, shops, socializes, etc. in a neighborhood with similar characteristics. Further, this presents less of a problem for our analysis than if we had studied rail ridership, whose stations generally draw riders from larger catchment areas (Daniels and Mulley 2013).

Finally, our analysis considers transit boardings in light of census tract characteristics; we do not assume or suggest that characteristics of these census tracts directly reflect the characteristics of transit riders in the tracts. This is because matching census data to transit boardings raises the spectre of ecological fallacy (Piantadosi et al. 1988). This is especially important when analyzing transit use, because transit riders tend to comprise relatively small shares of all travelers (Manville et al. 2022). An observer thus errs in assuming that any particular transit rider boarding in a given neighborhood necessarily resembles residents of that neighborhood. Lacking data about individual riders, our analysis cannot directly assess how rider characteristics changed during the pandemic. Further, while many bus trips begin or end close to a traveler’s home, about half of all transit trips begin or end in a neighborhood other than the traveler’s home neighborhood. While we also incorporate some tract data on worker characteristics from the LODES, we note as well that many transit boardings occur near neither the workplace nor the home. So, while we present and discuss the pre-pandemic characteristics of transit riders on each of the three systems analyzed, our multivariate analysis does not include data on transit rider characteristics. Instead, our analysis allows us to examine the characteristics of neighborhoods that lost the fewest and most riders during the pandemic.

## Results

We present here our analyses of the relationships between bus ridership change by census tract, neighborhood demographics, and built environment characteristics across the three regions during the pandemic. We establish differences in pre-pandemic bus use across the three transit operators and then transition to our analyses of ridership and demographics.

### Demographics of pre-pandemic bus riders

Boston, Houston, and Los Angeles are three very different cities in three distinct parts of the U.S. Unsurprisingly, their bus transit systems and the riders they serve vary as well. Data from pre-pandemic rider surveys cast these differences into sharp relief. Transit riders as a group differ substantially from non-riders in the U.S. And among transit riders, the travel modes (bus, subway, commuter rail, etc.) tend to serve different demographics. As noted above, U.S. bus riders prior to the pandemic tended to have lower incomes and are more likely to be people of color than rail riders (Taylor and Morris 2015).

**Fig. 2:**
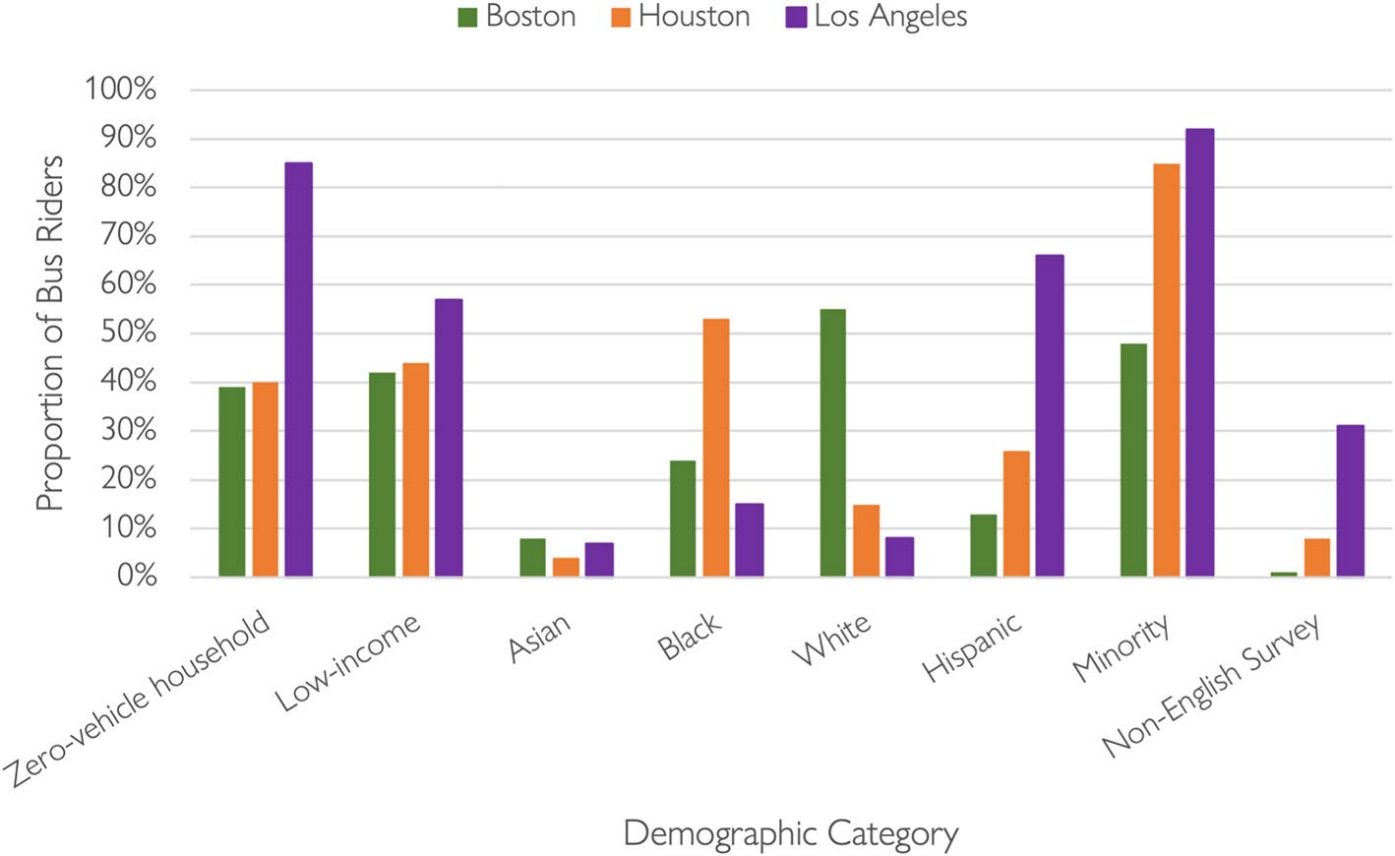
Bus rider characteristics in Boston, Houston, and Los Angeles circa 2015–2019. Data source: (Houston–Galveston Area Council 2018; LA Metro 2019c; MBTA 2018)

Boston is the oldest of the three cities, and a large share of its urban form developed in the nineteenth century when most people traveled by foot, bus, or streetcar. Houston, by contrast, is the youngest of the three, and most of its region developed around automobile travel. Los Angeles sits between the two: while more densely populated than many outside the Southland might imagine,^1^ Los Angeles is more auto-oriented than many big cities in the northeastern U.S., Chicago, and San Francisco. Compared to either Houston and Los Angeles, Boston has the oldest legacy public transit system, including the first subway in the U.S., and has a dense, transit-friendly center. Figure 2 compares ridership demographics on the three systems, drawing on pre-pandemic readership surveys conducted between 2015 and 2019.

In the 2010s, the majority (55%) of MBTA bus riders were non-Hispanic white and the majority (58%) lived in non-low-income households. In contrast to both Houston and Los Angeles, no single racial/ethnic group dominated the category of non-White (or “minority”) riders. Finally, almost none (1%) of the MBTA respondents took the agency-administered survey in a language other than English (MBTA 2018).

On Houston Metro, a majority (53%) of pre-pandemic bus riders were Black, and nearly twice as many (26%) were Hispanic than in Boston (13%). Despite these significant racial/ethnic differences, the share of bus riders from zero-vehicle households (39% in Boston, 40% in Houston) and low-income households (42% in Boston, 44% in Houston) were similar. Eight percent of respondents in Houston completed the survey in a language other than English (Houston–Galveston Area Council 2018).

In sharp contrast with the MBTA and Houston Metro, on LA Metro a substantial majority (66%) of pre-pandemic bus riders identified as Hispanic, and an overwhelming majority (85%) lacked private vehicle access. In addition, a substantially larger share of bus riders (57%) resided in low-income households (with incomes below 150% of the poverty line) than in Boston or Houston. While the racial/ethnic composition of bus riders varied substantially between Houston and Los Angeles, the total shares of racial/ethnic minority bus riders on the two systems (85% in Houston, 90% in Los Angeles) were similar, and well above the 48 percent in Boston. The proportion of LA’s bus riders who identified as Black was relatively small (15%) but was almost double the total share (8%) of African American people residing in Los Angeles County (U.S. Census Bureau 2019a, b). Finally, almost a third (31%) of LA Metro bus riders completed the survey in a language other than English, a rate substantially higher than on the other two systems (LA Metro 2019c).

### Ridership and neighborhood demographics

While overall bus ridership dropped dramatically in all three cities during the pandemic (as in all other U.S. regions), the magnitude of these declines—and rates of recovery—differed. Trends in Boston again show some divergence from the other two agencies and potentially greater challenges for the MBTA in recovering ridership post-pandemic.

#### Trends in bus ridership

**Fig. 3:**
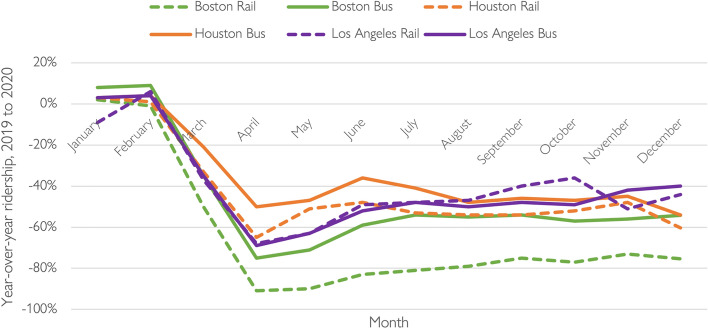
Year-over-year ridership by agency and mode, 2019–2020. *Data source* (Federal Transit Administration 2020)

Beginning in March 2020, overall transit ridership plummeted in all three regions. MBTA’s agency-wide decline (86%) was substantially larger than either Houston’s (58%) or LA’s (68%) declines. Figure 2 shows that rail ridership fell further and recovered more slowly than bus ridership in both Boston and Houston, while the pandemic collapse and mid-pandemic recovery of rail and bus riders in Los Angeles were roughly similar. However, Fig. 3 does not show the relative roles played by rail and bus in the three cities. At the MBTA, two-thirds of 2019 trips were on rail, and slightly less than a third were on buses. In contrast, two-thirds of 2019 Houston Metro trips and three-quarters of 2019 LA Metro trips occurred on buses (Federal Transit Administration 2020).

**Fig. 4:**
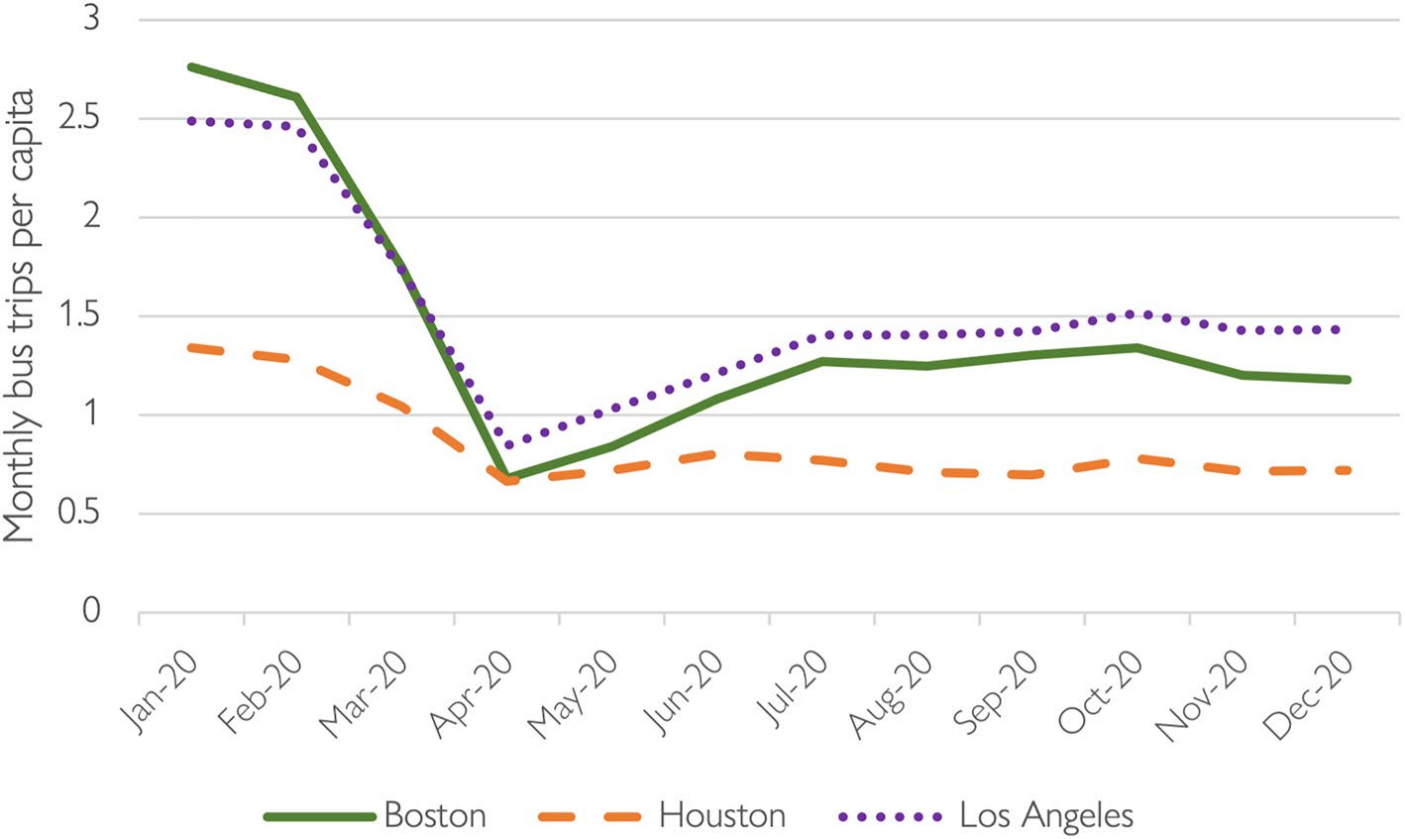
Monthly per capita bus ridership by region, 2020. *Data source* (Federal Transit Administration 2020)

The focus of our analysis is on bus use. Figure 4 shows that monthly *per capita* bus ridership was similar in Boston and Los Angeles before and during the pandemic, and significantly higher than in Houston for most of the periods analyzed. Because of their higher rates of pre-pandemic per capita bus ridership, the MBTA and LA Metro had more trips-per-rider to lose during the collapse than Houston Metro did.

#### Characterizing ridership declines by neighborhood

**Fig. 5:**
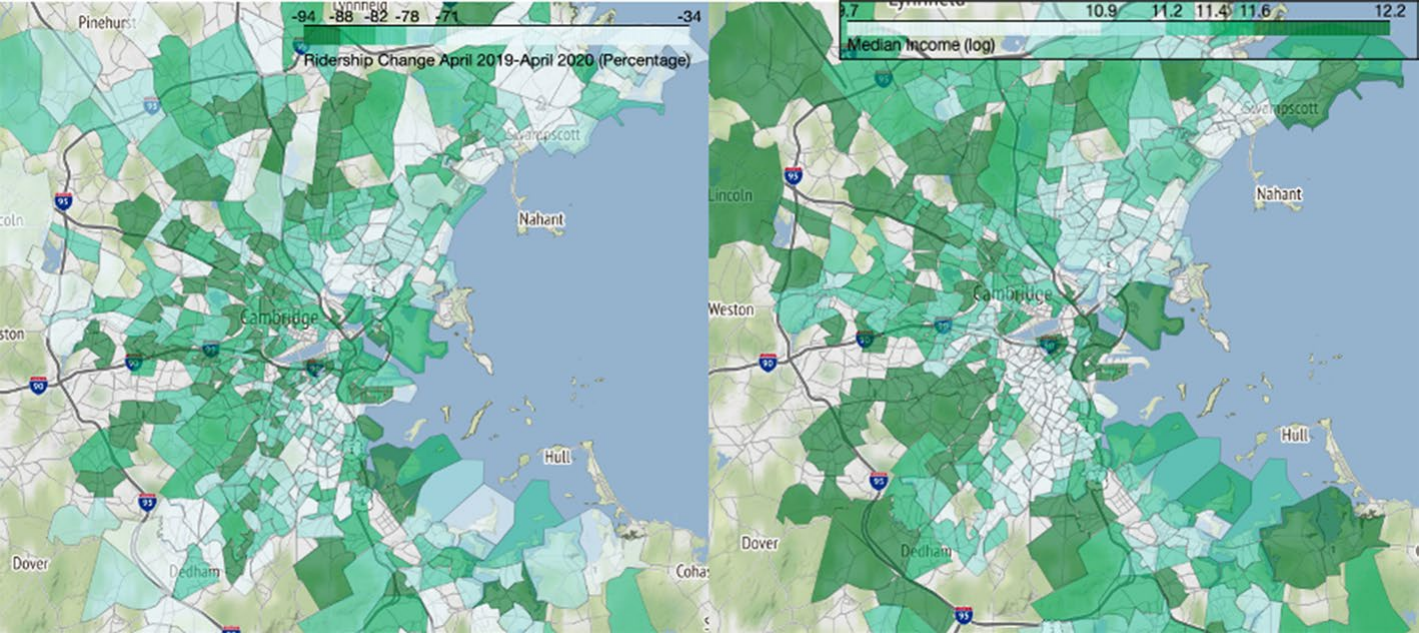
April 2019–2020 bus ridership change (left) and median household income (right), Boston metropolitan area. *Data source* (MBTA 2019a, 2020a; U.S. Census Bureau 2018). *Note* Income data are in natural log form

**Fig. 6:**
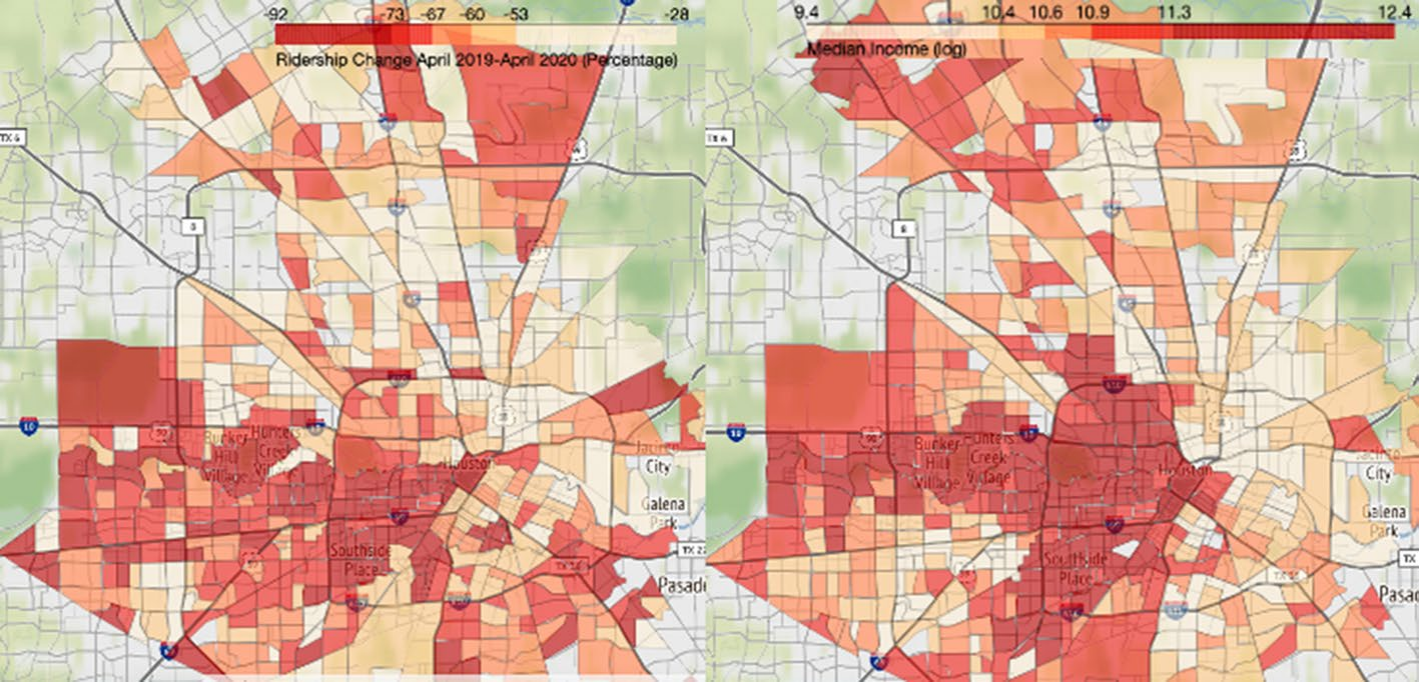
April 2019–2020 bus ridership change (left) and median household income (right), Houston metropolitan area. *Data source* (Houston Metro 2019a, 2020a; U.S. Census Bureau 2018). *Note* Income data are in natural log form

**Fig. 7:**
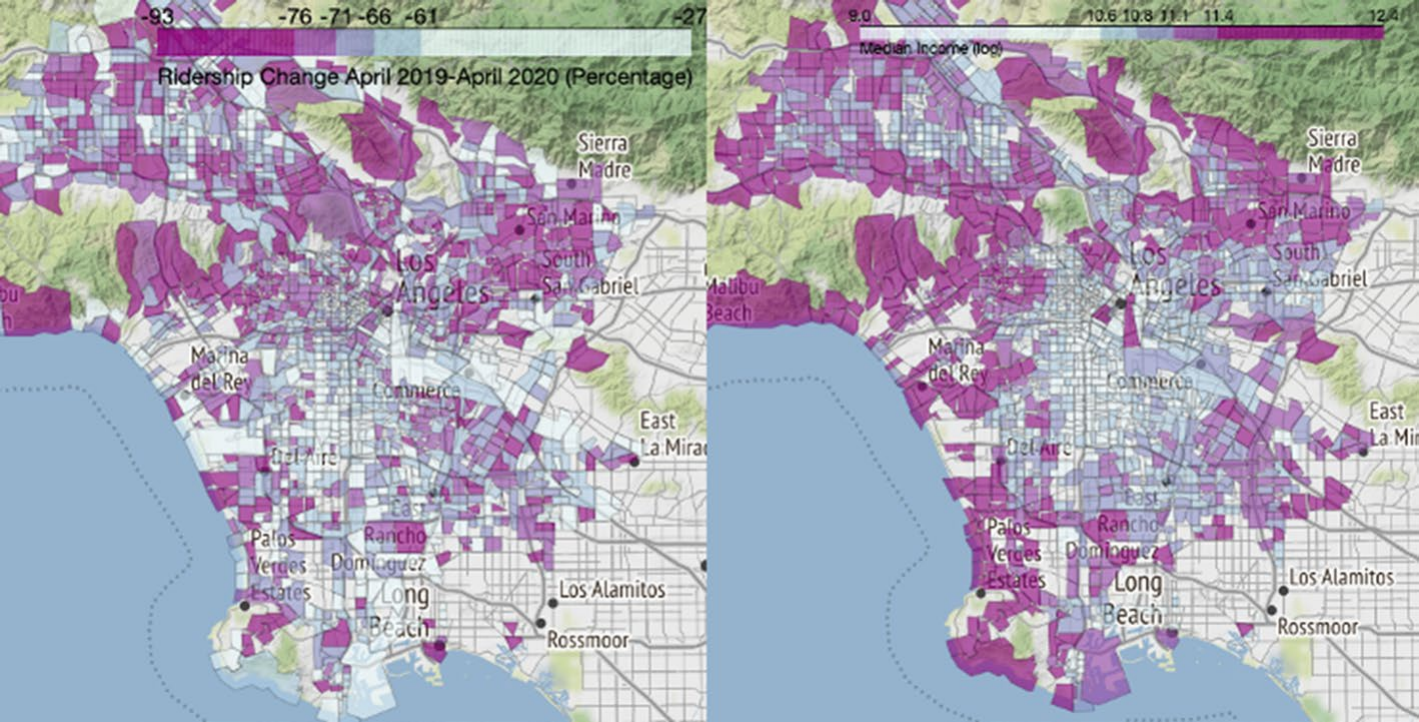
April 2019–2020 ridership change (left) and median household income (right), Los Angeles County. *Data source* (LA Metro 2019a, b; U.S. Census Bureau 2018). *Note* Income data are in natural log form

In addition to the top-line ridership figures presented above, we also analyze changes at the census tract level. Transit use is spatially asymmetric: a few of the oldest, largest cities host the most transit riders, and within metropolitan areas, a relatively small number of census tracts—such as in downtowns or low-income neighborhoods with lower average levels of auto access—host an outsize share of regional transit trips. Our analysis of the relative change in bus boardings does not reflect absolute differences across tracts, but instead on the relative change from pre-pandemic ridership levels (no matter how high or low those levels might be). Accordingly, Figs. 5, 6, and  7 show geographic patterns of the average rates of weekday ridership change in our three cities between April 2019 and April 2020 (the nadir of ridership), alongside a similar map showing 2014–2018 median household income (in natural log form).

These three map pairs illustrate two patterns. First, higher income neighborhoods tended to see larger proportional drops in bus ridership than lower income neighborhoods, though exceptions to this general pattern occur in each region; Pearson’s correlation coefficients between the log of median income and April 2019–2020 change in ridership are −0.274 for Boston, −0.239 for Houston, and −0.125 for Los Angeles. Second, bus ridership fell dramatically in the CBDs of each metropolitan area. These changes correspond with the documented drops in downtown office employment during the pandemic. In the multivariate analyses below, we examine each of these patterns further.

Table 1 shows the average changes in bus ridership across all neighborhoods in each region. Boston’s weekday bus ridership fell the most from April 2019 to 2020 (dropping 78%), while Houston fared better (dropping “only” 59%); Los Angeles landed in between (down 67%). While the same ranking remained by October 2020 (with Houston showing the smallest declines, followed by Los Angeles and then Boston), the gaps between regions had shrunk. Comparing neighborhood change from the beginning of the pandemic to mid-pandemic, we see that Boston’s average neighborhood bus ridership had recovered substantially. Average MBTA weekday bus ridership by neighborhood more than doubled between the two periods.

**Table 1:** Average change in weekday bus ridership by census tract, 2019 to 2020. *Data source* (Houston Metro 2019a, b, 2020a, b; LA Metro 2019a, b, 2020a, b; MBTA 2019b, a, 2020a, b)

Time period of change	Boston	Houston	Los Angeles
April 2019–2020	−78%	−59%	−67%
October 2019–2020	−59%	−50%	−52%
April 2020–October 2020	+ 169	+ 53%	+ 60%

#### Characteristics of top and bottom quintiles by region

**Fig. 8:**
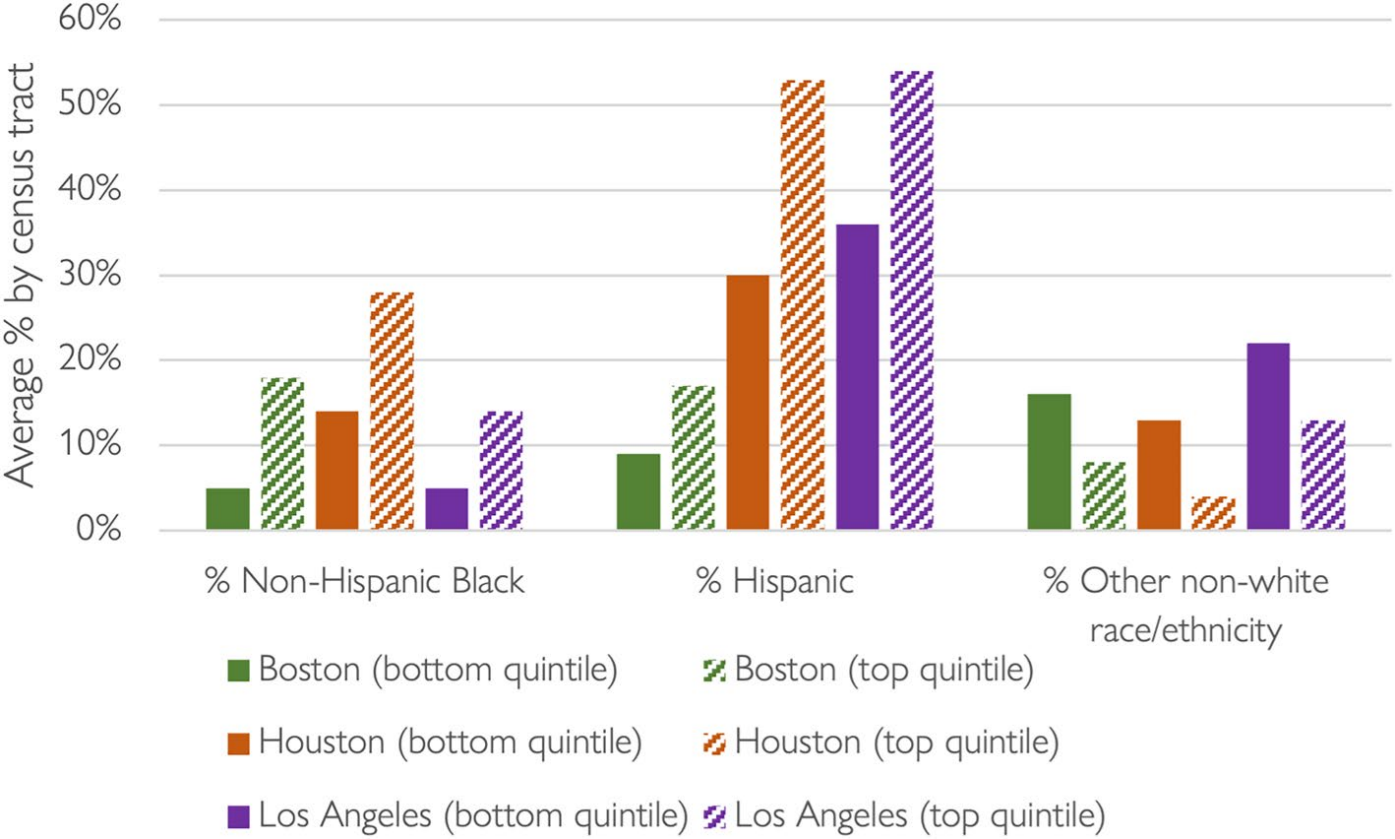
Racial/ethnic composition of the neighborhoods that lost the most (bottom quintile) and least (top quintile) shares of bus riders in Boston, Houston, and Los Angeles, April 2019 to 2020. *Data source* (Houston Metro 2019a, b, 2020a, b; LA Metro 2019a, b, 2020a, b; MBTA 2019b, a, 2020a, b; U.S. Census Bureau 2018)

While virtually all neighborhoods in the three metro areas lost ridership during the pandemic, Figs. 5, 6, and 7 show that drops in patronage were far from spatially uniform. To explore these shifts with respect to race/ethnicity and other socio-economic factors, we ranked the tracts in each region by relative weekday ridership loss between April 2019 and April 2020. Figure 8 compares the average residential demographics of tracts in the bottom (lost the most riders) and top (lost the fewest riders) quintiles.

While the demographics of Boston, Houston, and Los Angeles vary considerably from one another, in all three metropolitan areas census tracts that lost the largest shares of riders were home to fewer non-Hispanic Black and Hispanic residents than the tracts that lost the smallest shares. We also completed, but do not show due to space limitations, similar analyses for neighborhood ridership change between October 2019 and October 2020. While the patterns are generally the same, the gaps between the top and bottom quintiles of census tracts are narrower with respect to the proportion of African American residents.

**Fig. 9:**
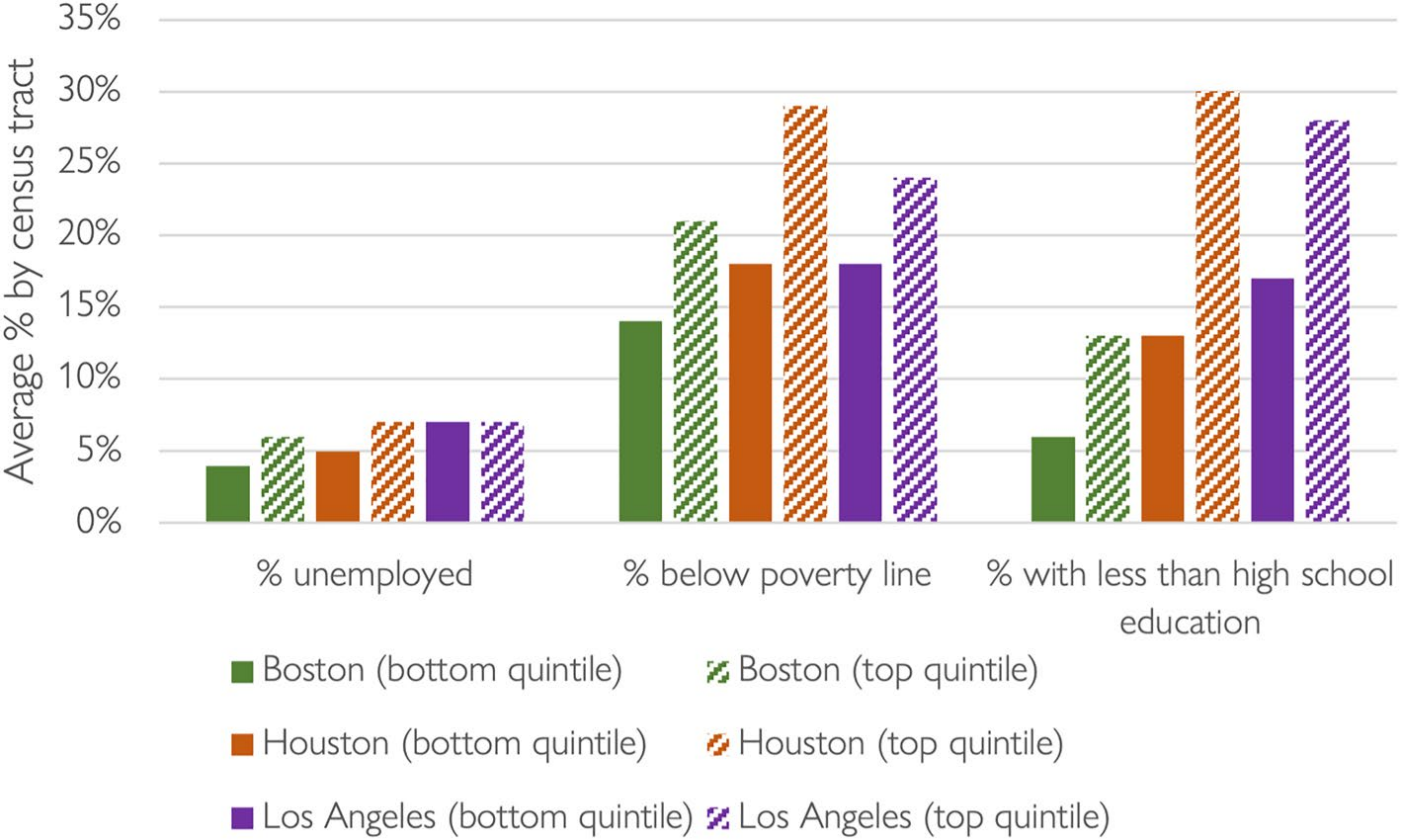
SES composition of the neighborhoods that lost the most (bottom quintile) and least (top quintile) shares of bus riders in Boston, Houston, and Los Angeles, April 2019 to2020. *Data source* (Houston Metro 2019b, a, 2020a, b; LA Metro 2019a, b, 2020a, b; MBTA 2019b, a, 2020a, b; U.S. Census Bureau 2018)

Figure 9 presents data for three tract-level socio-economic status (SES) variables across the three metropolitan areas: percent unemployed, percent in poverty, and percent of adults with less than a high-school education. Compared to the neighborhoods that lost the largest shares of riders, residents of the neighborhoods that lost the smallest shares of riders had slightly higher rates of pre-pandemic unemployment and much higher rates of households in poverty and adult residents with without a high school education. These effects were most stark in Houston (across all SES factors). By October 2020 (data not shown), the observed gaps between the top and bottom quintile neighborhoods generally shrank, but neighborhoods that lost the smallest shares of riders still had higher average levels of unemployment and poverty—as well as lower levels of education—than neighborhoods that lost the largest shares of riders.

### Modeling determinants of ridership change using neighborhood characteristics

We now turn to the relationship between ridership changes and neighborhood characteristics by combining agency-supplied data with census data. As before, our outcome of interest is the percentage change in ridership by census tract. Table 2 presents summary statistics for all census tracts in our analysis across the three regions.^2^

**Table 2:** Summary of key socioeconomic and built environment variables by region, 2019 to 2020. *Data source *(Houston Metro 2019a, b, 2020a, b*; *LA Metro 2019a, b, 2020a, b*; *Manduca 2020*; *MBTA 2019a, 2019b, 2020a, 2020b; U.S. Census Bureau, 2018, 2019b)

	Boston	Houston	Los Angeles
*Economic*			
Unemployment rate	6%	6%	7%
Below poverty line	19%	26%	22%
Less than high school education	11%	24%	25%
*Race/ethnicity*			
Non-Hispanic White	60%	24%	25%
Non-Hispanic Black	13%	23%	9%
Hispanic	15%	45%	50%
Other non-white race/ethnicity	12%	8%	16%
*Travel*			
Workers who commute via transit	23%	4%	8%
Households with vehicle available	79%	92%	89%
Workers able to WFH (by workplace location)	35%	32%	34%
*Built environment*			
Population density (persons/mile^2^)	14,831	6,429	15,416
Average miles to central business district (CBD)	6.51	9.14	10.78
*Bus ridership*			
April 2019 Average Weekday Tract Boardings	770	396	536
April 2020 Average Weekday Tract Boardings	170	131	182
October 2019 Average Weekday Tract Boardings	822	421	572
October 2020 Average Weekday Tract Boardings	345	184	263
Census tracts (n)	501	514	1,664

The data in Table 2 show that Boston, Houston, and Los Angeles are very different regions, and this goes for their transit systems as well. The Los Angeles sample includes over three times as many neighborhoods as either of the other two regions; this reflects both the enormous size of the Los Angeles region and the fact that LA Metro’s bus system serves a larger core service area than do Houston Metro or MBTA. Further, MBTA buses serve a slightly denser and less sprawling region than the other two transit agencies. In addition, both Houston and Los Angeles have larger proportions of non-White, poor, and low skill-workers living in their neighborhoods than does Boston. And in terms of travel behavior and auto access, Boston has a higher average transit commute mode share and smaller share of zero-vehicle households than the other regions. In 2019, Boston also had the highest average rates of bus ridership by neighborhood, in spite (or perhaps because) of the region’s extensive rail transit system and large numbers of rail riders.

Table 3 presents the results from our first set of OLS models. The outcome variable is the average percentage change in weekday bus boardings by neighborhood, weighted by neighborhood population. Each column presents the results of models for three time periods: Model 1 predicts year-over-year ridership change from April 2019 to 2020 (or early pandemic), Model 2 from October 2019 to 2020 (or mid-pandemic), and Model 3 from April to October 2020 (or early to mid-pandemic recovery).^3^ The pseudo-R-squared values indicate that their explanatory power ranges from 0.101 for the October 2019-to-October 2020 model to 0.268 for the April 2020-to-October 2020 model. We calculated Variance Inflation Factors (VIFs) for the variables in our models and found little evidence of multicollinearity (with a mean VIF of 2.46); we also found that the residuals are normally distributed for all our models.

**Table 3:** OLS models predicting percent change in average weekday bus ridership by neighborhood, 2019 to 2020. *Data source* (Houston Metro 2019a, b, 2020a, b; LA Metro 2019a, b, 2020a, b; Manduca 2020; MBTA 2019b, a, 2020a, b; U.S. Census Bureau 2018, 2019b)

	Model 1	Model 2	Model 3
	% Change in weekday ridership from April 2019 to April 2020	% Change in weekday ridership from October 2019 to October 2020	% Change in weekday ridership from April 2020 to October 2020
*Region (reference: Boston)*			
Houston	0.126***	0.029*	−0.672***
	(0.012)	(0.014)	(0.052)
Los Angeles	0.103***	0.017	−0.733***
	(0.011)	(0.012)	(0.047)
*Economic*			
Unemployment rate (%)	−0.032	0.139	0.479
	(0.089)	(0.101)	(0.376)
Below poverty line (%)	0.026	0.003	−0.491**
	(0.038)	(0.043)	(0.162)
*Race/ethnicity*			
Hispanic (%)	0.096***	0.085***	−0.206***
	(0.014)	(0.015)	(0.057)
Non-Hispanic Black (%)	0.125***	0.007	−0.591***
	(0.019)	(0.022)	(0.080)
Other non-white race/ethnicity (%)	−0.024	−0.072**	−0.084
	(0.023)	(0.026)	(0.096)
*Travel*			
Workers commuting via transit (%)	0.006	−0.024	−0.169
	(0.045)	(0.051)	(0.191)
Households with vehicle available (%)	−0.126**	−0.122*	−0.197
	(0.047)	(0.053)	(0.202)
Workers able to WFH (%)	−0.111***	−0.230***	0.011
	(0.024)	(0.028)	(0.104)
*Built environment*			
Miles to CBD	0.002***	0.003***	−0.004^
	(0.001)	(0.001)	(0.002)
Natural log, population density	−0.027***	−0.001	0.092***
	(0.004)	(0.005)	(0.018)
Tract share of total ridership	−1.757	−0.814	−49.125***
	(3.142)	(3.573)	(13.273)
Constant	−0.455***	−0.430***	0.931***
	(0.066)	(0.075)	(0.281)
Observations	2,614	2,627	2,596
R-squared	0.228	0.101	0.268

Standard errors in parentheses, ***p < 0.001, **p < 0.01, *p < 0.05, ^*p* < 0.10

While these models control for a variety of census-tract-level economic, racial/ethnic, travel behavior, and built environment characteristics, the regional dummy variables in each model are mostly statistically significant. In the models of tract-level early pandemic (April 2019 to April 2020) weekday ridership change, neighborhoods in Boston—home to by far the largest share of pre-pandemic public transit commuters among the three metropolitan areas—lost more riders than either Houston or Los Angeles (*p* < 0.001), all else equal. Houston neighborhoods also lost relatively fewer riders than Boston mid-pandemic (October 2019 to 2020), all else equal (*p* < 0.05).

With respect to the socio-economic characteristics of neighborhood residents, tracts with higher rates of poverty recovered fewer riders between April and October of 2020 (*p* < 0.01), but otherwise neither poverty nor unemployment rates were statistically significantly related to ridership changes.^4^ With respect to race/ethnicity, tracts with higher proportions of Hispanic residents lost fewer riders both early-(April 2020 to April 2019) and mid- (October 2020 to October 2019) pandemic (all *p* < 0.001), all else equal. Because such tracts lost relatively fewer riders early on, they had proportionally fewer riders to recover. So tracts with higher proportions of Hispanic residents added relatively fewer riders between April and October of 2020, *ceteris paribus*. The patterns were similar—fewer riders lost early in the pandemic (April 2019 to 2020), and fewer riders recovered between April and October of 2020—in neighborhoods with higher proportions of Black residents. In contrast to Hispanic neighborhoods, however, the proportion of Black residents was not related to mid-pandemic (October 2019 to 2020) ridership changes, while neighborhoods with higher proportions of other (non-white) race/ethnicities saw greater mid-pandemic declines in transit use, all else equal. These tract-level socio-economic and racial-ethnic correlates with pandemic ridership performance suggest that riders traveling to and from neighborhoods with higher proportions of white residents abandoned public transit at higher rates during the pandemic than in places with higher proportions of non-white travelers. However, changes over time do indicate nuanced effects.

As expected, neighborhoods with higher levels of private vehicle access lost a greater proportion of their pre-pandemic riders at both the beginning and mid-pandemic, which aligns with previous research on rates of mode-switching (from transit to driving) in 2020. Similarly, neighborhoods featuring workplaces in which a greater proportion of workers could work from home also lost more transit riders in both periods, all else equal. This suggests that the dramatic increases in working from home during the COVID-19 pandemic were indeed linked to falling transit use.

With respect to built-environment characteristics, census tracts closer to the CBD lost a greater share of riders at both the beginning and mid-pandemic than those further away, *ceteris paribus*. Similarly, higher density tracts lost a greater share of riders than low density tracts in the early- (April 2020) pandemic period, all else equal, yet this effect was no longer present by October 2020. Because in high-density neighborhoods trip origins and destinations tend to be closer together, it is possible that in these neighborhoods walking and cycling trips replaced transit travel at the start of the pandemic.

We also estimated 33 similar regression models (see Tables A1–A3 in the Appendix), by starting with the regional dummy variables, and then sequentially adding independent variables based on the magnitude of their standardized coefficients in the full OLS models. While the regional dummy variables remained statistically significant in most of the models, the magnitude of their effects were not the largest among the independent variables in the October 2020 to 2021 models: percent Hispanic, ability to work from home, tract distance from the CBD, and percent zero-vehicle households all ranked above the regional dummies in those models (though, similar to the models shown in Table 3, the explanatory power of the October 2019 to 2020 models were the lowest of the three time periods analyzed). Across all the models, other independent variables were associated with a substantial portion of their explanatory power. This suggests that these factors influenced transit irrespective of region-specific conditions. For example, in Model 1 in Table 3, the R^2^ increased from 0.154 (regional dummy variables only) to 0.228 in both the eight-variable parsimonious model and the 13-variable full model. Finally, we estimated three additional models wherein we replaced the regional dummy variables with the regional COVID-19 caseloads per capita for each metro area in April and October of 2020, as shown in Fig. 1 above (recall that caseloads in Boston were much higher than in Houston and Los Angeles early on). However, the magnitude of the COVID-19 caseload variable and the overall model R^2^ in these models (not shown) were lower in each of the three models compared with the models shown in Table 3.

To examine whether the effects of these independent variables were generalizable across the three regions, we estimated 39 additional variants on Models 1, 2, and 3 in Table 3, in which we interacted the regional dummy variables with the other independent variables. We present six of these models that interact our two built environmental variables (distance to the CBD and log of residential population density) with the Houston and Los Angeles regional dummy variables in Table 4 below. The other 33 models yielded few insights and are not presented due to space limitations (but are available upon request).

**Table 4:** OLS models predicting percent change in average weekday bus ridership by neighborhood with built environment interactions, 2019 to 2020. *Data source* (Houston Metro 2019a, b, 2020a, b; LA Metro 2019a, b, 2020a, b; Manduca 2020; MBTA 2019a, b, 2020a, 2020b; U.S. Census Bureau 2018, 2019b)

	Model 4	Model 5	Model 6	Model 7	Model 8	Model 9
	% Change in ridership from April 2019 to 2020	% Change in ridership from April 2019 to 2020	% Change in ridership from October 2019 to 2020	% Change in ridership from October 2019 to 2020	% Change in ridership from April to October 2020	% Change in ridership from April to October 2020
*Region (reference: Boston)*						
Houston	0.191***	0.089	0.173***	−0.394***	−1.029***	0.748^
	(0.024)	(0.097)	(0.027)	(0.109)	(0.102)	(0.412)
Los Angeles	0.173***	−0.141^	0.158***	−0.510***	−0.840***	−0.671*
	(0.019)	(0.074)	(0.021)	(0.084)	(0.080)	(0.314)
*Economic*						
Unemployment rate (%)	−0.034	−0.043	0.137	0.135	0.425	0.386
	(0.089)	(0.089)	(0.100)	(0.100)	(0.375)	(0.376)
Below poverty line (%)	−0.024	−0.004	−0.100*	−0.080^	−0.363*	−0.412*
	(0.040)	(0.040)	(0.045)	(0.045)	(0.168)	(0.169)
*Race/ethnicity*						
Hispanic (%)	0.098***	0.097***	0.089***	0.091***	−0.237***	−0.225***
	(0.014)	(0.014)	(0.015)	(0.015)	(0.058)	(0.058)
Non-Hispanic Black (%)	0.130***	0.131***	0.021	0.028	−0.649***	−0.618***
	(0.019)	(0.019)	(0.022)	(0.022)	(0.081)	(0.081)
Other non-white race/ethnicity (%)	−0.017	−0.017	−0.056*	−0.056*	−0.150	−0.087
	(0.023)	(0.023)	(0.026)	(0.026)	(0.097)	(0.096)
*Travel*						
Workers commuting via transit (%)	0.041	0.008	0.055	0.001	−0.291	−0.234
	(0.046)	(0.045)	(0.051)	(0.051)	(0.194)	(0.192)
Households with vehicle available (%)	−0.183***	−0.166**	−0.237***	−0.235***	−0.082	−0.085
	(0.049)	(0.050)	(0.055)	(0.056)	(0.208)	(0.213)
Workers able to WFH (%)	−0.107***	−0.108***	−0.221***	−0.231***	−0.007	0.040
	(0.024)	(0.024)	(0.027)	(0.028)	(0.104)	(0.104)
*Built environment*						
Miles to CBD	0.010***	0.002***	0.019***	0.003***	−0.024***	−0.003
	(0.002)	(0.001)	(0.002)	(0.001)	(0.008)	(0.002)
Natural log, population density	−0.023***	−0.042***	0.006	−0.044***	0.079***	0.135***
	(0.004)	(0.008)	(0.005)	(0.008)	(0.018)	(0.032)
Share of total ridership	−1.252	−1.949	0.185	−1.216	−50.382***	−49.172***
	(3.133)	(3.135)	(3.532)	(3.547)	(13.237)	(13.240)
Interactions (reference: variable * Boston)						
Miles to CBD * Houston	−0.007**		−0.016***		0.038***	
	(0.002)		(0.002)		(0.010)	
Miles to CBD * Los Angeles	−0.008***		−0.017***		0.015^	
	(0.002)		(0.002)		(0.008)	
Natural log, population density * Houston		0.004		0.049***		−0.165***
		(0.011)		(0.013)		(0.047)
Natural log, population density * Los Angeles		0.027***		0.059***		−0.010
			(0.009)		(0.034)	
Constant	−0.497***	−0.281**	−0.522***	0.057	1.128***	0.442
	(0.066)	(0.096)	(0.075)	(0.108)	(0.285)	(0.407)
Observations	2,614	2,614	2,627	2,627	2,596	2,596
R-squared	0.234	0.232	0.123	0.115	0.274	0.273

Standard errors in parentheses, ***p < 0.001, **p < 0.01, *p < 0.05, ^*p* < 0.10

Table 4 suggests some interesting statistically significant relationships. First, the positive relationship between distance from the CBD and ridership losses—i.e., that more outlying tracts saw smaller declines (see Models 1 and 2)—was weaker in both Houston and Los Angeles than in Boston for both time periods, all else equal. Indeed, in a stratified model of Houston alone (not shown here), the relationship between ridership and distance from CBD was not statistically significant in any of the models for any of the different time periods. Second, higher population density tracts in Los Angeles lost relatively fewer riders by both April and October of 2020 than did similar tracts in Boston, all else equal; the same was true for the less-dense Houston in relation to Boston, but only for the October 2019 to 2020 period.

Overall, Models 4 through 9 perform similarly to those without the interaction terms. Yet like Models 1 through 3, they also have relatively modest R-squared values, ranging from 0.115 to 0.274. Such R-squared values are common in studies of travel behavior, given that many factors that affect travel—such as personal preferences, life experiences, situationally-specific circumstances, and even physical fitness—are not captured in large-scale surveys like the ACS.

## Discussion and conclusion

This analysis examines the neighborhood-level correlates with bus ridership change in three very different U.S. regions during the first year of the COVID-19 pandemic. To our knowledge, this is the first article to examine the influence of neighborhood level factors on ridership change across multiple metropolitan areas.

Our findings first suggest that while pre-pandemic bus transit riders were disproportionately people of color, low-income, and from households without a car, these differences between bus riders and the general population may have grown even starker during the early months of the COVID-19 pandemic (as measured by the neighborhoods where riders boarded buses). But while some of the associations between neighborhood socio-economic and race/ethnicity variables and transit use weakened as the pandemic wore on, many remained significant, nonetheless. Such changes likely reflect the constraints that travelers in disadvantaged neighborhoods faced in adjusting their travel behaviors, relative to those in better-resourced neighborhoods.

Second, regional differences in bus ridership losses and recovery—such as MBTA’s decline (86%) dwarfing that of Houston Metro’s (58%) at the start of the pandemic (Federal Transit Administration 2020)—can at least partly be attributed to the different services they provide and markets they serve. In terms of regional effects, Boston—with its whiter and comparatively more affluent rider base, many of whom were likely able to shift to remote work or from riding the bus to driving—lost the largest share of riders at the beginning of the pandemic, compared to Houston and Los Angeles. Neighborhoods in MBTA’s core service area also have higher population densities than Los Angeles and especially Houston, which may have presented Bostonians with more opportunities to replace transit trips with walking. But even after controlling for an array of socio-economic and environmental neighborhood-level factors, the regional effect continued to exert a strong influence on ridership change in almost all our models.

The robust regional effects in our models imply underlying metro-specific effects that our suite of independent variables did not capture. For example, transit agency actions likely played a role. A parallel analysis to this one of transit service changes in the pandemic found that the scale of bus service changes were largest in Boston, less in Los Angeles, and very small in Houston (Dasmalchi and Taylor 2022). Other regional differences may be at play as well. For example, it is possible that early in the pandemic Houston’s public officials better communicated the relative safety of riding public transit than their counterparts in Los Angeles and Boston. Similarly, the timing and duration of the stay-at-home orders, as well growth in infection and hospitalization rates, may have played a role as well. Indeed, in our models that replaced regional dummy variables with COVID-19 caseloads per capita in each region, the caseload variable was statistically significant in each case. It may also be that the late summer and early fall heat and humidity of Houston could have motivated more travelers to ride transit rather than walk or cycle, in comparison with the more temperate summers of Boston and Los Angeles.

Third, we find an association between neighborhood race/ethnicity composition and bus ridership change, even when controlling for SES factors like poverty rates and built environment factors like population density and centrality. This suggests an important relationship between race/ethnicity and transit use apart from these other factors. Bus riders boarding in neighborhoods with higher shares of Hispanic residents were more likely to remain riding both early- and mid-pandemic, *ceteris paribus*. Bus riders boarding in neighborhoods with higher shares of Black residents were more likely to remain riding early in the pandemic, all else equal, but by October this differential effect disappeared.

Fourth, neighborhoods with more alternatives to riding transit in the pandemic saw greater patronage losses. Tracts with higher proportions of residents with a car available to them, and those with workplaces with higher proportions of potential work-from-home employees, were both associated with larger ridership drops in both the early- and mid-pandemic periods.

Fifth, the built environment effects on neighborhood ridership change varied by region. In general, urban centrality had a negative effect on bus ridership. This relationship held particularly true in Boston; however, the effect was weaker in Los Angeles and ambiguous in Houston. Similarly, population density—while positively correlated with transit use generally—was associated with proportionally larger ridership losses in both our April 2019 to 2020 and October 2019 to 2020 models. Further, this effect was stronger in densely developed Boston than in Los Angeles or comparatively low-density Houston. These two findings likely reflect regional differences in urban spatial structure and the polycentric nature of auto-oriented cities like Houston and Los Angeles, both of which host many decentralized sub-centers of commercial activity.

Public transit in the U.S. serves two principal markets: (1) people traveling to dense agglomerations of activity (like downtowns) where driving is difficult and parking expensive, and (2) travelers who, because of age, income, or ability, have little or no access to private vehicles (Garrett and Taylor 1999). This first market largely collapsed during the COVID-19 pandemic, while the second market moved to the forefront. While transit ridership fell nearly everywhere in the pandemic, it fell furthest in cities (like Boston) and in districts (like downtowns) where more educated, affluent, and white riders could work from home or drive instead of riding transit.

While transit use has slowly recovered at this writing (in the summer of 2022), late-pandemic riders continue to board disproportionately in less advantaged neighborhoods. It appears that “choice” riders—or at least people who travel to or from places where more “choice” riders live—may return to transit more slowly than constrained ones. Working from home appears likely to persist, at least part-time, for many office workers; this may reduce employment densities in CBDs and other employment centers traditionally served by transit (Haag 2021; Liu and Su 2021). And demand for suburban and exurban housing in large metropolitan areas has outpaced urban housing demand during the pandemic, particularly in the largest, most transit-friendly metropolitan areas (Dougherty and Casselman 2021). Meanwhile, rising demand for private vehicles during the later stages of the pandemic has combined with pandemic-related supply-chain disruptions to create shortages of new cars; prices of used cars and trucks rose 45 percent between June 2020 and June 2021, the largest one-year increase ever recorded (The Economist 2021).

It may be that surplus downtown office space and lower rents will eventually attract firms with workers more inclined to ride transit than those they replace. And it may be that residents who decamp urban areas for the suburbs will be replaced by less affluent and more transit-friendly urbanites. And it may be that high vehicle and fuel prices will prevent frequent transit users from acquiring and driving the more affordable vehicles they seek. But none of these outcomes is assured. Further, each could play out in a manner—fewer downtown workers and central city residents with more access to and use of cars—that bode ill for transit.

But what does appear assured is that the least advantaged travelers with lower rates of private vehicle access and limited abilities to work from home will continue to depend on public transit for mobility, perhaps more than ever in the coming months and years. Effectively serving these riders will entail deploying (and perhaps redeploying) transit service in less advantaged neighborhoods where demand is highest and needs are greatest.

## Supplementary Information

Below is the link to the electronic supplementary material.

Supplementary file1 (XLSX 19 kb)

## Footnotes

1 The Los Angeles Urbanized Area (UZA) is the most densely populated in the U.S. (U.S. Census Bureau, 2019a, b).

2 The data in Table 2 reflect averages for census tracts in each region (weighted by population), and not region-wide averages. Further, our analysis includes all the census tracts served by buses operated by the region’s largest transit operator; it does not include all census tracts in each metropolitan area.

3 Note that compared to Models 1 and 2 (pre-pandemic to pandemic), Model 3 examines ridership changes from early to mid-pandemic – as indicated by Table 1 (i.e., modest ridership gains rather than steep losses) – and the signs on the coefficients should be interpreted with this in mind. The same is true of Models 8 and 9 as compared to Models 4 to 7 (presented below).

4 With respect to neighborhood education levels, we ultimately excluded our percent of tract residents with a high school diploma from our models because it was very highly correlated with the work-from-home potential variable we discuss below.
